# 2.D. Workshop: The challenges of developing population health intervention research in cancer health promotion

**DOI:** 10.1093/eurpub/ckac129.072

**Published:** 2022-10-25

**Authors:** Organised by: French National Cancer Institute, Chair persons: Jérôme Foucaud (France)

## Abstract

As the leading cause of death in Europe and the second leading cause of death in North America, it is known that about 40% of cancers are preventable. Until recently, it was considered that if we know the risk factor, then we can act on it: experience has shown that this knowledge is necessary but insufficient to know how to act. Population health intervention research (PHIR), a ‘solution’ science, addresses this challenge. It is concerned with the design, implementation, evaluation, adaptation, transferability and durability of interventions aimed at improving the health of populations, in order to produce valid knowledge with a high potential for social and health impacts. This scientific approach apprehends interventions as ‘events in systems’ and challenges the methodological hegemony imposed for many years by the biomedical sciences.

Today, the development of PHIR in health promotion in the domain of cancer faces three major challenges:

– Policy;

– Methodology;

– Transferability.

Three complementary presentations are proposed, each of which will respond directly to these challenges and each of which will be based on concrete examples of implementation in the domain of cancer. The aim of this workshop is to offer a time for exchange and discussion on the challenges to be met in developing PHIR in health promotion, in the domain of cancer and at all stages of the disease, as an innovative model of research within an innovative paradigm shift. It will enable participants to clarify their conceptions of PHIR for the advancement of health promotion in oncology and to evolve these concepts in relation to these three key issues. An interactive format is proposed, including time for discussion based on participants’ perceptions and time for presentations.

**Key messages:**

• PHIR: an effective scientific approach and a relevant tool for thinking about and developing health promotion policies in the field of cancer.

• a new research paradigm for cancer control based on intervention with populations to act on health determinants.

